# An Efficient Data Partitioning to Improve Classification Performance While Keeping Parameters Interpretable

**DOI:** 10.1371/journal.pone.0161788

**Published:** 2016-08-26

**Authors:** Kristjan Korjus, Martin N. Hebart, Raul Vicente

**Affiliations:** 1 Computational Neuroscience Lab, Institute of Computer Science, University of Tartu, Tartu, Estonia; 2 Department of Systems Neuroscience, Universitätsklinikum Hamburg-Eppendorf, Hamburg, Germany; National Taiwan University, TAIWAN

## Abstract

Supervised machine learning methods typically require splitting data into multiple chunks for training, validating, and finally testing classifiers. For finding the best parameters of a classifier, training and validation are usually carried out with cross-validation. This is followed by application of the classifier with optimized parameters to a separate test set for estimating the classifier’s generalization performance. With limited data, this separation of test data creates a difficult trade-off between having more statistical power in estimating generalization performance versus choosing better parameters and fitting a better model. We propose a novel approach that we term “Cross-validation and cross-testing” improving this trade-off by re-using test data without biasing classifier performance. The novel approach is validated using simulated data and electrophysiological recordings in humans and rodents. The results demonstrate that the approach has a higher probability of discovering significant results than the standard approach of cross-validation and testing, while maintaining the nominal alpha level. In contrast to nested cross-validation, which is maximally efficient in re-using data, the proposed approach additionally maintains the interpretability of individual parameters. Taken together, we suggest an addition to currently used machine learning approaches which may be particularly useful in cases where model weights do not require interpretation, but parameters do.

## Introduction

The goal of supervised machine learning, in particular classification, is to find a model that accurately assigns data to separate predefined classes. To test the generality of a learned model, this model is typically applied to independent test data, and the accuracy of the prediction informs a researcher about the quality of the classifier [1]. Finding a classifier that performs optimally according to the researcher’s objective requires a set of assumptions and also a trade-off in model complexity: Too simple parameters lead to under-fitting, i.e. the model is not able to account for the complexity of the data. Too complex parameters at the same time lead to over-fitting, i.e. the model is too complex and fits to noise in the data. To test different assumptions and also optimize this so-called bias-variance trade-off [2], it is quite common to divide a data set into three parts: (1) a training set and (2) a validation set, which together are used iteratively for optimizing the parameters of the chosen classifier, and (3) a separate test set to validate the generalization performance of the final classifier.

Machine learning methods in life sciences are used with different objectives: At one end of the spectrum, the goal is making predictions in real-world applications and building a maximally predictive model with interpretable weights and parameters that can be used in future applications. At the other end, the goal is to make an inference about the presence of information, where even the slightest discrimination performance indicates a statistical dependence between independent and dependent variables (e.g. classes and data). In the latter approach, the interpretability of weights and parameters and their reuse is not the focus of the research and performance is commonly evaluated using statistical analyses (e.g. [3]). This latter approach is quite common in the field of neuroimaging [4] and bioinformatics [5].

**Fig 1:**
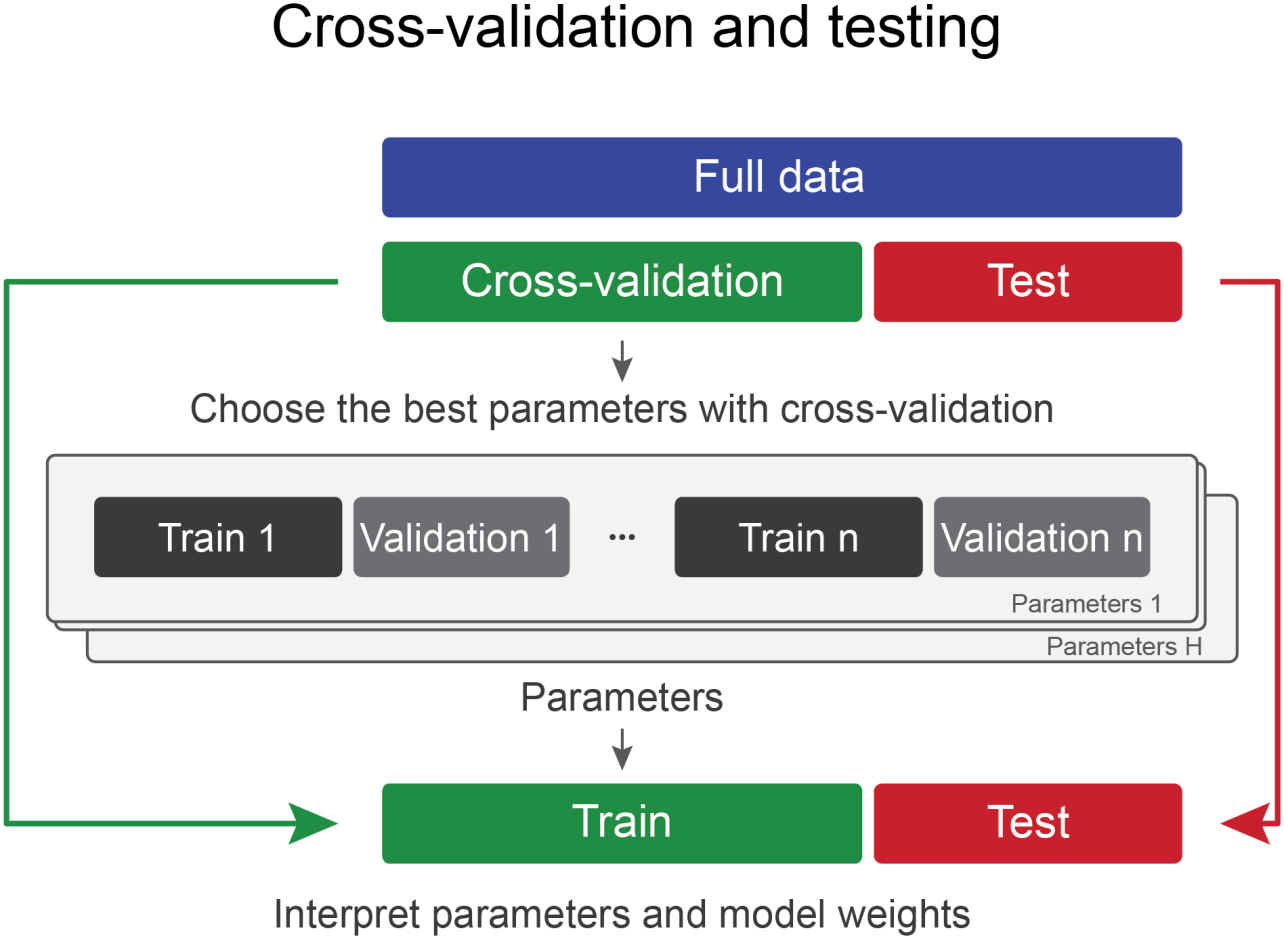
In the “Cross-validation and testing” approach, the data are divided into two separate sets (cross-validation set and test set) only once. First, different models are trained and validated with cross-validation and the best set of parameters is chosen. Prediction accuracy and statistical significance of the parameters are evaluated on the test set, after training on the cross-validation set.

Data collection can be very expensive in biological and social sciences, and over time more data-efficient methods have emerged [6]. Cross-validation is a method that makes near-optimal use of the available data by repeatedly training and testing classifiers on different subsets of data, typically with a large training and a small validation set in each iteration [7]. For example, in 5-fold cross-validation 80 percent of the data are used for training, 20 percent for validation, and in the next iteration another 20 percent of data are chosen as a test set, etc. This process is repeated five times until all data have served as validation data once. Cross-validation is repeated with different parameter combinations, and once the best parameters have been found, the model is trained with the chosen parameters on all data that have previously been used for cross-validation and applied to the separate test set (see Fig 1). When the goal of a researcher is to build a model that generalizes well to unseen cases and that may be used in real life applications such as image recognition or text classification, this approach is probably the most generally used method for carrying out classification analyses, with five to ten fold cross-validation resulting in a good trade-off between over-fitting and sufficient training size of the classifier [8].

One difficulty of this approach is that the test set that is used to validate classification performance is limited in size. While cross-validation makes good use of training data, the estimation of the generalization performance of the classifier may still suffer by this limited size of the final test set. Increasing the size of the test set at the same time would come at the cost of diminishing classification performance. When data are scarce or expensive to acquire, this can become a large problem and may lead to a sub-optimal choice of classifiers and the associated parameters.

**Fig 2:**
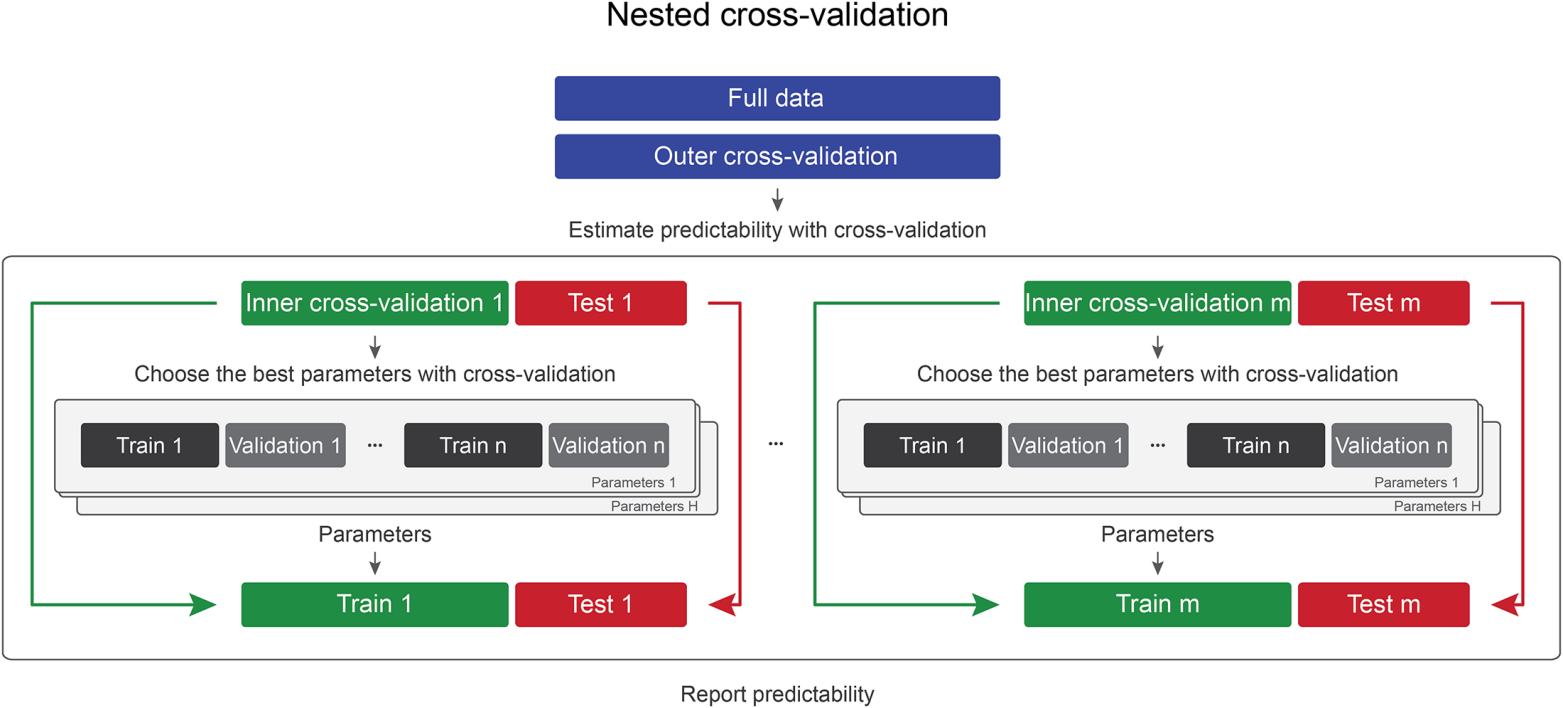
In the “Nested cross-validation” approach, first (outer) cross-validation is performed to estimate predictability of the data. In each iteration, data are divided into training and test sets. Before training, another (inner) cross-validation loop is used to optimize parameters. As model weights (fitted models) and parameters are different at every partition, it is not possible to report accuracy or statistical significance about a particular set of parameters or model weights.

One approach that has been used to overcome this difficulty is “Nested cross-validation” [9] (see Fig 2). Here, the test set is not kept completely separate, but cross-validation is extended to incorporate all available data (outer cross-validation). In that way, all data can serve as test set once, overcoming the aforementioned trade-off. In order to still be able to optimize parameters, for a particular cross-validation iteration the training set is again divided for nested cross-validation (inner cross-validation), and once the best parameters for this iteration have been found, they are used to train a model on the current training data, which is then applied to the current test set. This approach is most useful for a researcher who does not need to build a model that generalizes well to unseen data, but who would like to describe whether there is a meaningful statistical dependence between the class labels and the given dataset, in other words whether the dataset contains information about the labels.

While a Nested cross-validation procedure makes more efficient use of data, it has some drawbacks: Due to the absence of a completely separate test set it is not possible to claim that a particular model, i.e. a particular set of weights and classifier parameters, could in the future be used to classify unseen data [2]. In addition, the chosen parameters and models may vary between cross-validation iterations, making it impossible to select one set of parameters or one model as the final choice. In other words, a separate model and a separate set of parameters are chosen in each iteration and choosing any one of them would mean returning to a simple cross-validation and testing approach which would annihilate the advantage gained by nested cross-validation. There are, however, cases in which the interpretation of parameters is desirable, even when the model is not used. For example, for certain applications it might be useful to report that the best parameter corresponds to linear models as opposed to quadratic ones, without the need to describe the specific model weights used by the linear models. In another example, when using neural network as the class of machine learning algorithms, the number of layers selected during the optimization, say 3, 4 or 5 layers, may be an important choice to be communicated to other researchers and that may lend some interpretation to the best combination of parameters and data.

**Fig 3:**
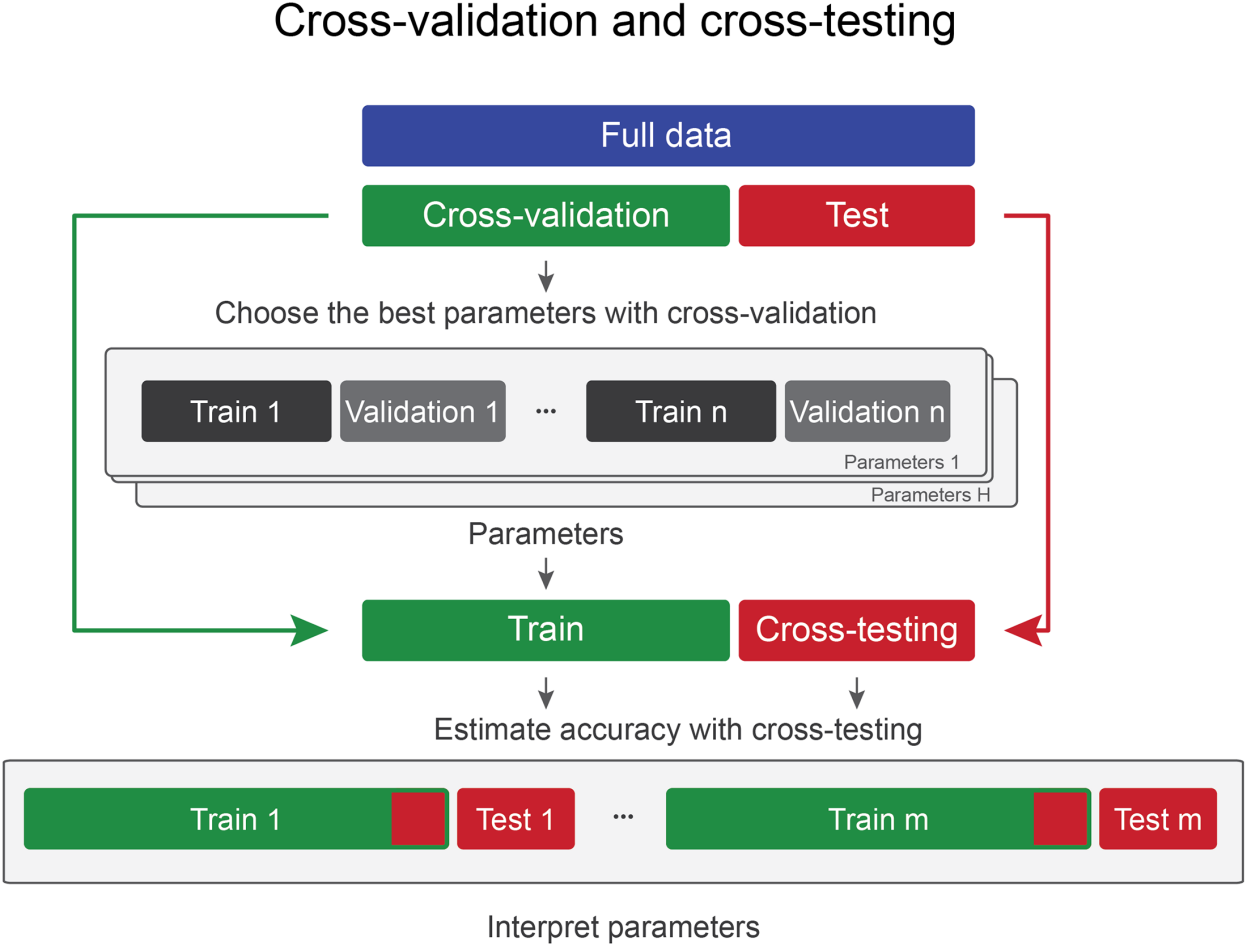
For cross-validation and cross-testing, data are divided into two separate sets only once: a cross-validation set and a test set. Similar to typical cross-validation, a number of iterations are carried out to choose the best parameters for the final model on the test set. Once the best combination of parameters has been chosen, the prediction accuracy and statistical significance can be evaluated on the test set with a modified cross-validation such that for each fold the original cross-validation set is repeatedly added to the training data. Due to the similarity to cross-validation, we term this approach cross-testing. While making it impossible to pick one final model on additional unseen data, the parameters that have been chosen remain interpretable.

Here we describe a novel approach that improves the trade-off between training and test size for better generalization performance than “Cross-validation and testing”, while, in contrast to “Nested cross-validation”, maintains the interpretability of chosen parameters. In the case of the widely used “Cross-validation and testing” approach, a larger test set results in less data for choosing the best set of parameters and also less data for fitting the model. In contrast, with our newly proposed “Cross-validation and cross-testing” approach (Fig 3) a larger test set still means that we are left with less data for parameter selection, but it does not reduce the amount of data available for model fitting. This comes at the cost of losing the ability to generate a single predictive model that is used in the future for general application. However, for many researchers it is sufficient to (1) demonstrate that information is indeed present in the dataset [4] or (2) interpret the parameters of the classifier. The latter can also guide a number of important choices for future design of classifiers. The novel approach suggested in this work improves the trade-off by using data more efficiently for fitting the model but makes it still possible to choose, interpret and report a set of parameters that may be used in the future. In brief, it is a mixture of the approaches of “Cross-validation and testing” and “Nested cross-validation”, which allows reusing test data to improve the size of the training set, thereby improving classification performance. We term this method “Cross-validation and cross-testing”.

The current article is structured as follows. In the methods section we will describe the data sets and parameters used in order to test the applicability of the proposed approach on simulated and real data. In the first part of the results section we will then outline the novel approach of “Cross-validation and cross-testing” in detail. In the second part of the results section, we will compare all approaches using numerical experiments described in the methods part and show that the novel approach has a superior classification performance than the common “Cross-validation and testing” approach. We also demonstrate that the novel pipeline is not biased by applying it to simulated data for which ground-truth is known.

## Methods

In the present work we compare three different approaches for machine learning analysis methods: “Cross-validation and testing”, “Nested cross-validation”, and the newly proposed method “Cross-validation and cross-testing”. We predict that in terms of generalization performance, “Nested cross-validation” will outperform both other methods, at the cost of making the chosen parameters and weights uninterpretable. “Cross-validation and testing” is predicted to generate the worst performance, while allowing interpretation of both optimal parameters and the weights of the final model for generating future predictions on additional unseen data. Finally, we predict that “Cross-validation and cross-testing” will have prediction performance between the other two approaches, yielding no interpretable model weights, but fully interpretable model parameters.

We will compare these different methods on four different data sets. Our approach is as follows. Each time, a subset of data is randomly sampled from a larger data set which serves as a reference data set. The three different methods are then evaluated using this reference data set. The mean classification accuracy is compared against chance, using a statistical cutoff of *p* < 0.05. This procedure is repeated 1,000 times and the proportion of significant results and the average accuracy are reported.

### Parameters

To illustrate our theoretical predictions, we applied the three analysis approaches for optimization of two parameters. Firstly, for pre-processing the choice was between no preprocessing or performing a dimensionality reduction using principal component analysis (PCA) [10] with 70% of cumulative variance preserved. Secondly, three possible values (0.0001, 0.01 and 1) can be chosen for the penalty for misclassification (“c”) of the Support Vector Machine (SVM) algorithm [11, 12]. The combination of the two parameters give rise to a total of 6 possible parameter combinations. To speed the computational time hereafter we restrict ourselves to the before-mentioned 6 parameter combinations but many other parameters can be considered such as the type of normalization of the data set or the different types of classifier models.

The k-fold parameter, the number of random partitions used in cross validations, is fixed to 5 in all the cases.

We would also like to note that the term *parameter* has been used inconsistently in the literature, sometimes referring to the individual weights of a given model, and sometimes to the parameters that are used to optimize the learning algorithm. Here, we use the term *parameter* to refer to a variable that is used to optimize classification performance (which may incorporate choices not directly applied to a particular classifier, including the choice of pre-processing or the choice of classifier). For parameters related to the model itself, we use the term *model weights*.

Please note that although our novel approach makes it possible to interpret the chosen parameters, the chosen parameters are not reported, for two reasons. First, they have been chosen quite arbitrarily for illustrative purposes only. Second, they have been repeatedly selected based on subsets of data that have been repeatedly sampled. For those reasons, we believe that interpretation of parameters in this context is not very meaningful.

### Size of the test set

The “Cross-validation and testing” and “Cross-validation and cross-testing” approaches both have an important hyper-parameter, size of the test set, that must be determined before proceeding to any further analysis.

With each of our 4 data sets, the trade-off between test and training data was varied in two different ways. First, we varied the size of the whole data set while fixing the test set proportion to 50%.

Second, we kept the total amount of data fixed and examined how the test set size affected the results. The size of the full data set was fixed between two extremes (always and never finding a statistically significant result) in order to illustrate the differences in the relative sizes of the test set. The size of the data set was fixed to 50 for EEG data and to 100 in all other cases. The following sizes for the test set were used: 10%, 30%, 50%, 70% and 90%.

### Datasets

We used four datasets, three of which contain information and are classifiable. The three classifiable datasets consist of a simulated data set and and two datasets from neural recordings. The neural datasets contain two types of electrophysiological data: electroencephalogram (EEG) and spiking data. The fourth dataset consists of randomly generated data with no classifiable information. A brief description of the datasets is given below. More information about the collection of the datasets can be found in the respective references.

All datasets can be found in the Supplementary Information, S1 Public Repository.

#### Simulated data

We generated 4,000 data samples of two classes each (A and B) with 6 dimensions (features) sampled from a uniform distribution between 0 and 1. For class A, 2,000 data points were left unchanged, but we added a scalar 0.8 to the first two dimensions of the remaining 2,000 data points. For class B, we added 0.8 to the first dimension of the first 2,000 data points and 0.8 to the second dimension of the remaining 2,000 data points. These modifications make the classes linearly inseparable.

The final generated dataset has 6 features with 8,000 data points from both of the two classes.

#### EEG data

The resting state EEG data analyzed here were collected prior to 12 different cognitive EEG experiments conducted at the University of Tartu. [13] The measurements took place in a dimly lit and quiet room. Participants sat in a comfortable office chair 1 or 1.15 m away from a computer screen. They were instructed to relax and avoid excessive body and eye movements. There were two conditions: eyes open and eyes closed. During the eyes open condition subjects were also required to fixate on a black cross in the middle of a gray screen. From the EEG time series the power spectral density values were computed using the Fourier transform with Hamming tapered 2 s windows.

The two labels of the data are “eyes open” and “eyes closed”. The features consist of 91 power spectral density values from 5 occipital electrodes (‘PO3’ ‘PO4’, ‘O1’, ‘Oz’, ‘O2’). The final “EEG dataset” consists of 455 features with 289 samples from two classes.

#### Spike train data

The neural spiking dataset contains multiple single unit recordings from different rat hippocampal and entorhinal regions while the animals were performing multiple behavioral tasks. In particular, we used a single session of a single rat from the CA1 region in hippocampus. The dataset (“hc3”) is accessible from the neural data repository crcns.org [14]. The data used includes spikes of 61 neurons, and we sampled 2,000 data points while the rat was engaged in active spatial navigation in a square-shaped arena. We divided the square into two areas to assign two types of labels to each neural recording data point according depending on whether at that moment the rat was at the “upper part” or “lower part” of the arena. In total, the “spike train dataset” has 61 features with 2,000 data points from two classes.

#### Random data

We generated 10,000 data samples with a random assignation to two classes. Each data point has 20 dimensions sampled from a uniform distribution between 0 and 1. Thus, the “random dataset” has 20 features with 10,000 data points from two classes.

### Statistical significance

In addition to reporting general classification accuracy for each of the datasets, we also report the proportion of results that were found to indicate accuracies significantly above chance. In the context of classification, statistical significance indicates whether a statistical dependence between labels and data can be assumed. A statistically significant results would reject the null hypothesis that the association of data and labels comes about by chance, and the p-value would indicate the probability of false rejection of the null hypothesis. Statistical significance was evaluated with a permutation test (number of permutations was fixed to 1,000 and statistical significance level to 0.05) [15].

It is worth noting that our datasets are sometimes very small and we have to repeat the permutation test many times. For that reason, it becomes important to correct the p-value for the bias introduced by the discrete nature of classification results as described in [16]. If in one permutation test the resulting p-value was in between two possible p-values around the significance level a probabilistic approach was used to determine if the given run was significant or not. Otherwise the nominal alpha level could never be approximated. The implementation of it can be found from the code in S1 Public Repository. The effect of the correction was small and did not change the results.

## Results

In this section, we will describe the novel approach that we term “Cross-validation and cross-testing”. In particular, we will explain the algorithm and compare it with two other common approaches known as “Cross-validation and testing”, and “Nested cross-validation”. We note that the proposed approach is a natural interpolation between the two classical approaches. In the second part of this section, we will apply the proposed pipeline to simulated data as well as biological datasets.

### Cross-validation and cross-testing

When a researcher is interested in publishing their model parameters, then typically the efficient and popular approach called “Cross-validation and testing” is used (Fig 1). In the cross-validation set, the best parameters are chosen—usually according to highest cross-validation accuracy—and the test set is used for out-of-sample accuracy estimation.

An even more data-efficient approach for data analysis is called “Nested cross-validation” (Fig 2). The approach is similar to cross-validation and testing, but the test set becomes part of an outer cross-validation loop, while parameters are optimized in inner cross-validation iterations, using only the training data of the current outer cross-validation iteration. The whole data set can be used for estimating the final accuracy and therefore has a maximum statistical power for significance analysis. However, this approach does not make it possible to publish parameters which might be sometimes desirable as discussed in the Introduction section.

Our proposed pipeline can be seen as a natural extension between the two extremes described before. See Table 1 for a comparison of the three approaches in terms of data efficiency and parameter and model weights interpretability.

**Table 1:** Comparison of the approaches.

Approach	Data efficiency	Possible to interpret parameters	Possible to interpret fitted model
Cross-validation and testing	Low	Yes	Yes
**Proposed**: Cross-validation and cross-testing	Intermediate	Yes	No
Nested cross-validation	High	No	No

Comparison of the newly proposed “Cross-validation and cross-testing”, classical “Cross-validation and testing” and “Nested cross-validation” with respect to data efficiency, and parameter and model interpretability.

“Cross-validation and cross-testing” starts by carrying out the common “Cross-validation and testing” approach: The best parameters are chosen with cross-validation as described previously. Once the parameters are fixed, the remaining data are used for testing the classifier. The novelty of the approach is introduced by how prediction accuracy and statistical significance are evaluated (see Fig 3). Rather than keeping the test set entirely separate, a modified training set is iteratively introduced, where in each iteration the original training data plus part of the originally separate test data are used for training a classifier, and the remaining test data for testing the classification performance. We term this iterative procedure “cross-testing”, because this term more accurately describes the process that is repeated than “cross-validation”. Importantly, this approach maintains independence between training and test data, but makes more efficient use of test data by augmenting training data for more accurate predictions. In the next iteration, a different part of test data is added to the training data, and this process is repeated until each part of the test data has once been added to training data. The mean prediction accuracy across these different cross-validation iterations is then averaged. We refer to this novel approach as “Cross-validation and cross-testing”.

This proposed approach is expected to provide more accurate results than classical “Cross-validation and testing” by construction because it simply uses more data for model fitting, while the system for choosing the best set of parameters remains the same.

### Simulated data

**Fig 4:**
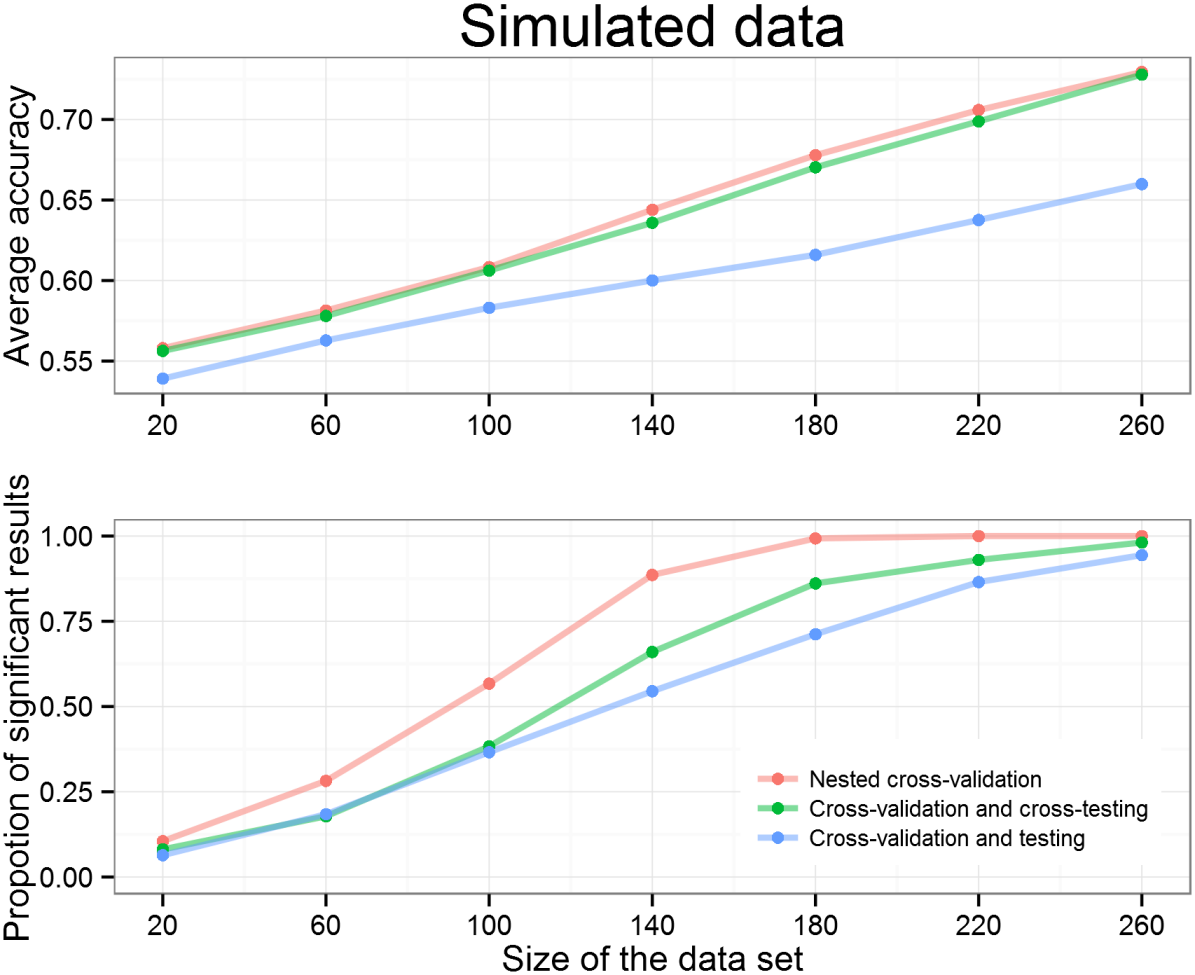
Batches of simulated data of different sizes are analyzed 1000 times with three different approaches. Results show the mean accuracy (upper plot) and proportion of significant results (bottom plot) out of the 1000 runs. More data leads to higher average accuracy and increases the proportion of significant results. “Nested cross-validation” outperforms other approaches and the “cross-validation and testing” gives the worst performance in terms of average accuracy and proportion of significant results.

To compare the three approaches we first analyzed the simulated data set containing two classes that are linearly inseparable. We start by varying the data set size while fixing the test set size at 50%. It can be seen from Fig 4 that larger data set size increases the average accuracy and the proportion of significant results. We can also see that “Nested cross-validation” provides a larger percentage of significant results. The approach using the proposed “Cross-validation and cross-testing” gives significant results more often that the popular “Cross-validation and testing” approach. Thus, while only “Cross-validation and testing” and the proposed approach “Cross-validation and cross-testing” make it possible to report parameters, the latter is more sensitive since more data is effectively used for model fitting.

**Fig 5:**
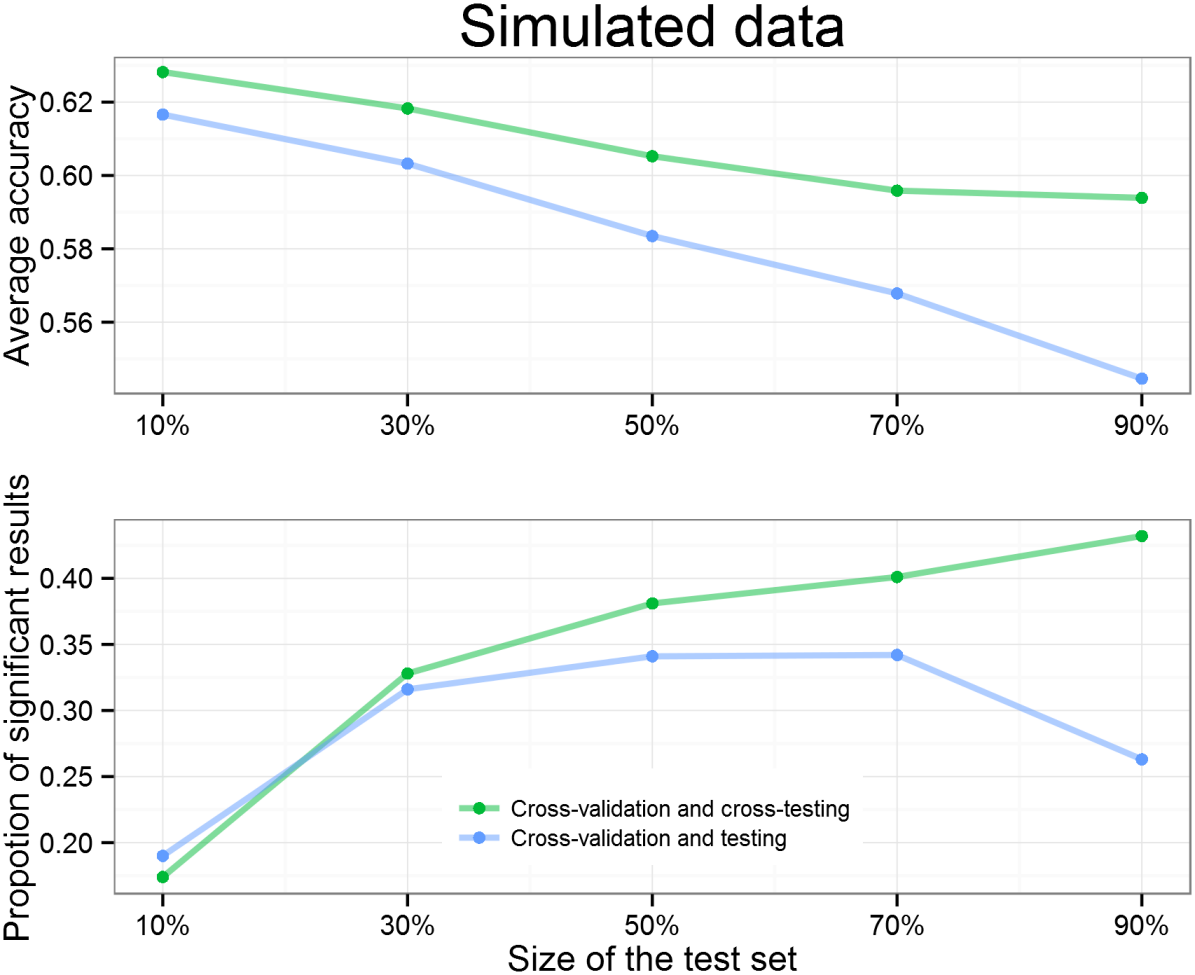
Batches of 100 simulated data points were analyzed 1,000 times with both approaches that contain a separate test set. Results show the mean accuracy (upper plot) and proportion of significant results (bottom plot) out of the 1000 runs. A larger test set leads to smaller average accuracy because there is less data for choosing parameters and fitting a model. “Cross-validation and cross-testing” outperforms “cross-validation and testing” in terms of average accuracy and proportion of significant results as expected.

The “Cross-validation and testing” and “Cross-validation and cross-testing” approaches are affected by the size of the separate test set, which must be determined before proceeding to any further analysis. Therefore, we tested the effect of the test set size by fixing the data set size at 100 data points and using different proportions of the 100 data points for testing. The clearly visible downward trend on the first graph in Fig 5 shows that using a larger test set decreases the average accuracy because there is less data for choosing the best parameters. The observed difference in average accuracy is due to the test data resampling. As expected, the higher average accuracy is also reflected in the bottom graph on the Fig 5 by more often leading to significant results.

Usually for the popular approach “Cross-validation and testing” it is recommended that the size of the test set should be about 10% to 50%. From the bottom graph on the Fig 5 we can see that for this case it happened that the optimal point is more close to 70%. Interestingly, the proposed approach “Cross-validation and cross-testing” has a different optimal size for the test set. As test data resampling changes the trade-off, we can observe from the bottom graph of Fig 5 that for our proposed approach a test set size around 90% would give a significant result with highest probability.

### Real data

**Fig 6:**
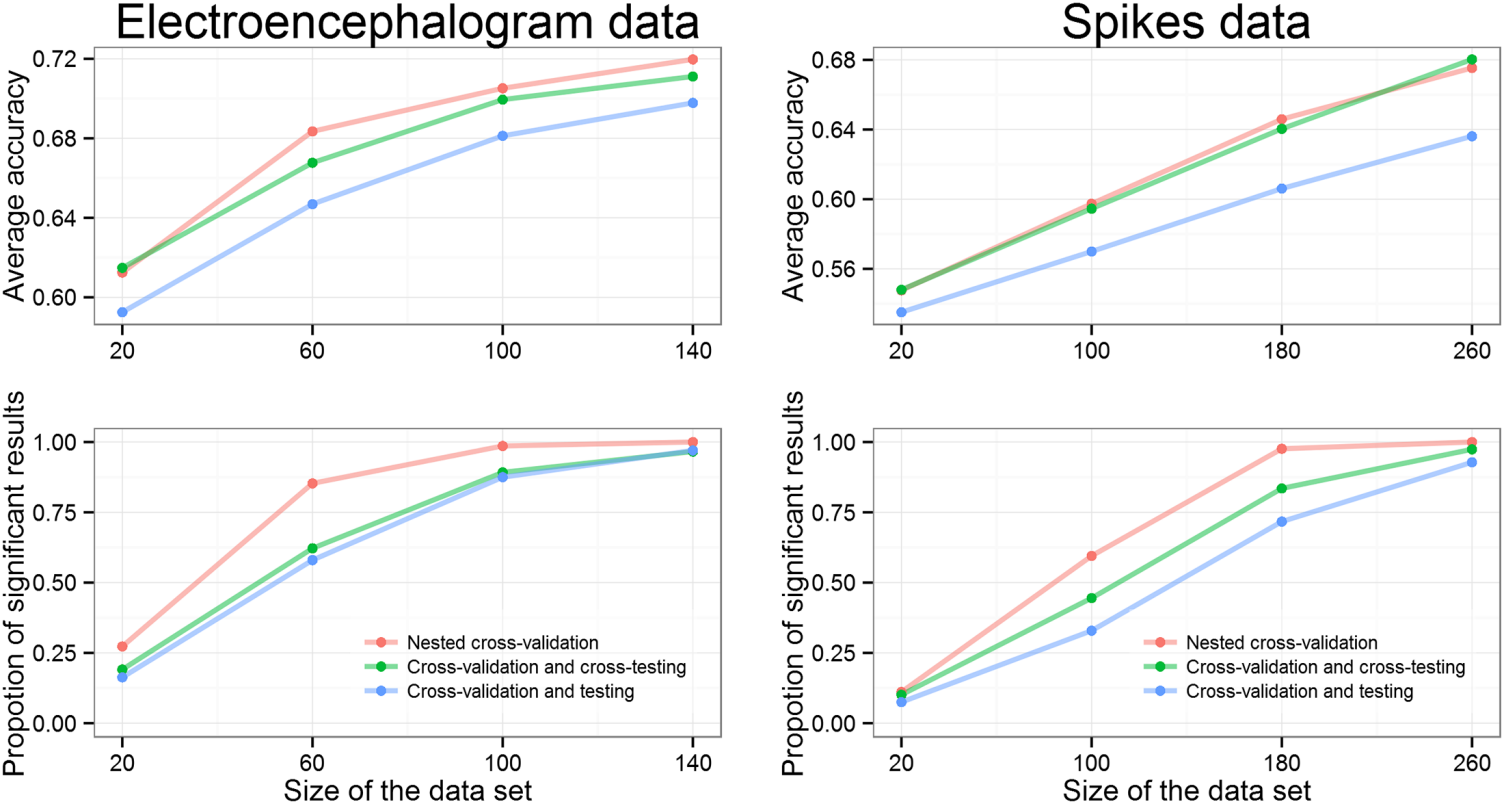
Analysis of real data (left: EEG dataset; right: spiking dataset) with three different approaches as a function of data size with test set size fixed at 50%. Results show the mean accuracy (upper graphs) and proportion of significant results (bottom graphs) out of the 1000 runs. More data lead to higher average accuracy and increases the proportion of significant results. “Nested cross-validation” outperforms other approaches while “Cross-validation and testing” gives the worst performance in terms of average accuracy and proportion of significant results. The effect is smaller with EEG data suggesting that more efficient usage of data in the model fitting is not that important and the choice of parameters is actually the main influencer.

We also analyzed two neuroscience datasets: EEG data from humans and spiking data from rat hippocampus. From Fig 6 we can observe the same trends as above: larger data set sizes imply higher average accuracy and more frequent significant results. We can also see that the approach “Nested cross-validation” gives significant results most often, followed by the proposed approach “Cross-validation and cross-testing”, which gives significant results more often than the popular “Cross-validation and testing” approach. The effect is smaller with EEG data suggesting that more efficient usage of data in the model fitting is not critical for this dataset (eyes open and closed conditions can be clearly distinguishable from few samples of EEG power spectra) and the choice of parameters is actually the main determinant for the classifier performance.

**Fig 7:**
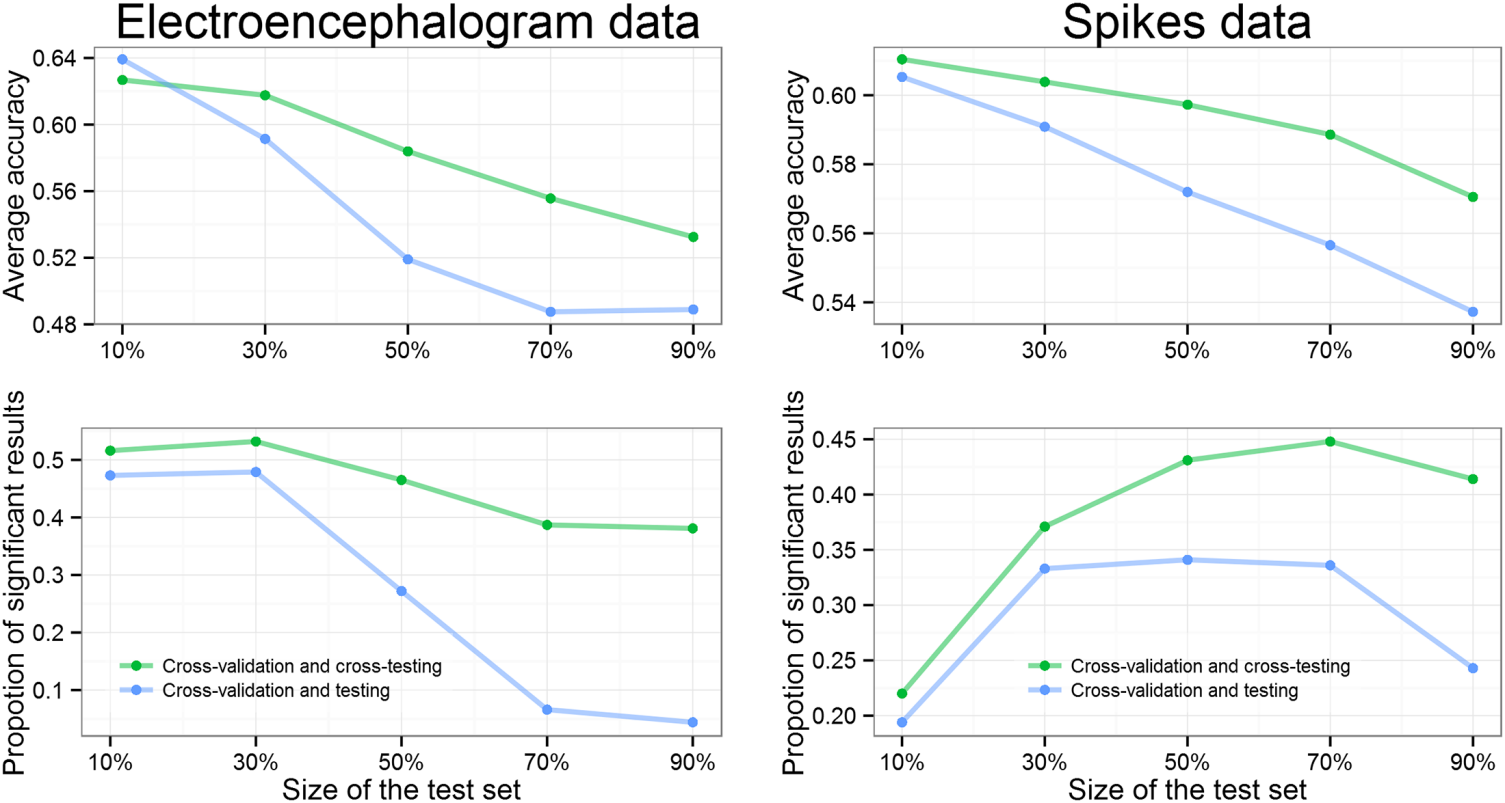
Analysis of neuroscience data (left: EEG dataset; right: spiking dataset) with three different approaches as a function of the relative test set size. Results show the mean accuracy (upper graphs) and proportion of significant results (bottom graphs) out of the 1000 runs. Data set size was fixed to 50 for EEG and to 100 for spikes train data set. Larger test set leads to smaller average accuracy because there is less data for choosing parameters and fitting a model. “Cross-validation and cross-testing” outperforms “cross-validation and testing” in terms of average accuracy and proportion of significant results.

Fig 7 shows that “Cross-validation and cross-testing” outperforms “Cross-validation and testing” in terms of accuracy and proportion of significant results for a large range of relative test set sizes. Also, as expected, larger test set size reduces the average accuracy because there are less data for choosing the parameters. Interestingly, optimal size for the test set for EEG data is about 30% for both approaches but for the spiking data optimal size for the test set changes from 50% to 70% if approach is changed from “Cross-validation and testing” to “Cross-validation and cross-testing”. We also note that in the limit of small test sets both approaches should converge to the same result on average. This explains the crossover of “Cross-validation and testing” and “Cross-validation and cross-testing” in the average accuracy of the electroencephalogram data (top left) where for very small data sets noise begins to dominate results.

### Random data

**Fig 8:**
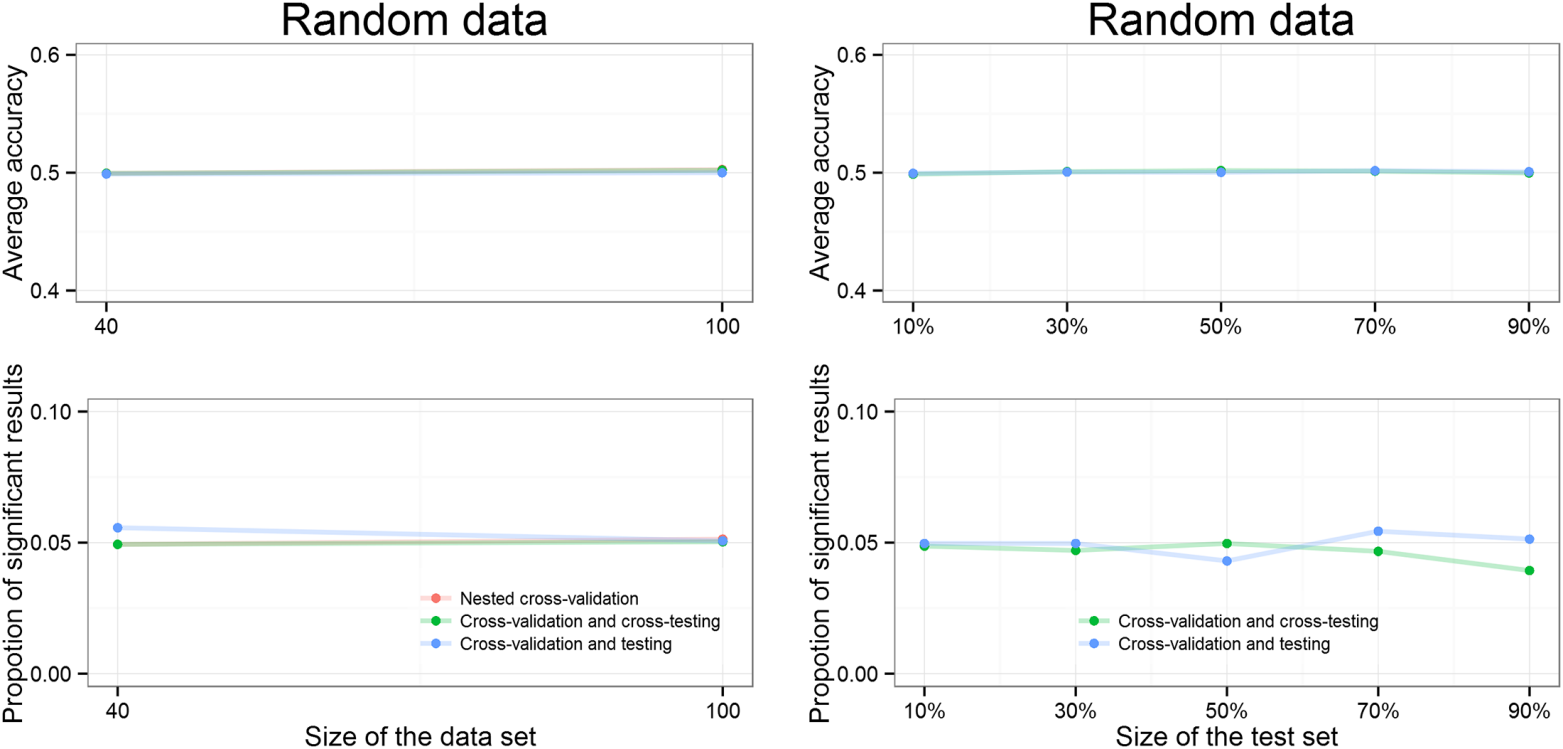
Accuracy and proportion of significant results are kept at chance levels when applying the novel approach to random data.

To validate our whole set-up we also tested it on noisy data which was randomly assigned to 2 classes, and thus no information should be classifiable for this dataset beyond chance level. As seen from Fig 8, the average accuracy is about 50% as expected for random data and the proportion of significant results stays around 0.05 which is also expected as we set of significance threshold to 0.05.

## Discussion and Conclusion

When using machine learning algorithms for making predictions, improving performance of a classifier can be seen as a central goal. At the same time, interpretability of models and parameters beyond the given data are in many cases desirable. Since data are often scarce or expensive to acquire, efficient use of data is another important objective. These three goals—generalization performance, interpretability, and efficient use of data—often lead to a trade-off that is resolved depending on the focus of the researcher. If the focus lies on generating a model that will generalize well to unseen data, this requires an interpretable model and interpretable model parameters. For that purpose, data can be used efficiently by the common approach known as “Cross-validation and testing”. If, however, the goal is to maximize classification performance for finding a statistical dependence between class labels and data, then a better performance can be achieved by using “Nested cross-validation”, which provides a very data efficient approach by repeatedly re-using data. However, this approach does not naturally allow the interpretation of parameters and model weights.

Previously, researchers had to choose from these two extremes depending on their goal. In this article, we described a novel approach that uses cross-validation to find and fix the best combination of parameters, but importantly which then resamples test data for augmenting training data, yielding to more accurate estimation of generalization performance. We termed this new approach “Cross-validation and cross-testing” and predicted that it would outperform outperforms “Cross-validation and testing” in terms of accuracy and statistical sensitivity with simulated and empirical data sets. In particular, we tested the effects of different data sets, data set sizes and test set sizes.

Indeed, we confirmed in various numerical experiments that too large test sets quickly result in insufficient data for finding the best parameters and not enough data for fitting the best model. On the other hand, too small test sets can imply that there are not enough data for achieving statistically significant results. This trade-off for the size of the test set results in the existence of an optimal range. The proposed “Cross-validation and cross-testing” modifies the range to allow larger test set sizes because with the novel cross-testing part it is still possible to use almost all of the data for model fitting.

As predicted, we demonstrate the superiority of “Cross-validation and cross-testing” over “Cross-validation and testing” both in terms of accuracy and in terms of statistical sensitivity. On simulated data where half of the data served as test data we demonstrated that “Cross-validation and cross-testing” performed similar to “Nested cross-validation” in terms of accuracy, but outperformed “Cross-validation and testing” irrespective of data size. This improved performance can be explained by the much larger data set that is available during each testing iteration of “Cross-validation and cross-testing”.

In terms of statistical significance, “Cross-validation and cross-testing” constantly outperformed “Cross-validation and testing”, but demonstrated particular sensitivity for intermediate data set sizes, demonstrating the particular usefulness of “Cross-validation and cross-testing” for small to intermediate data sets. Varying the test set size also demonstrated that larger test sets can be superior for the novel approach; however, this depends on the structure of the data and whether parameters can be estimated well even when using very little training data. It is not generally advisable to maximize the test set at the cost of the training set size.

Indeed, for empirical neurophysiological data we found similar results as for simulated data in terms of accuracy and statistical sensitivity. Again, “Cross-validation and cross-testing” generally outperformed conventional “Cross-validation and testing”. Here, however, the test set size trade-off did not favor a maximally large test set for the novel approach, but varied between the different data sets. The EEG classification of eyes open and eyes closed resulted in a high accuracy and displayed a sweet spot between the limits of little training data and little test data, with a significant improvement compared with the classical cross-validation and testing approach. The neuronal spiking data from hippocampus is much more variable and probably non-linearly separable with the features given but exactly the same trends were observed with respect to the dependency of the different approaches on training and test sizes. In general, for the empirical data sets used in this article and for maximizing statistical sensitivity, the optimal test set size was around 50%. However, additional simulations and analyses are required before recommendations for maximal performance can be made. This, however, is beyond the scope of the present work.

Finally, as a sanity check the analyses were repeated on random data to demonstrate that “Cross-validation and cross-testing” did not introduce any statistical bias. Indeed, the proportion of significant results was around the expected level of significance, demonstrating that the novel approach was not statistically biased.

While both “Cross-validation and testing” and the proposed “Cross-validation and cross-testing” make it possible to report parameters, the latter, novel approach uses data more efficiently in the final model fitting phase by reducing the test set size trade-off. This change of the trade-off occurs by reducing the detrimental effect of a larger test set size on the quality of the fitted model by augmenting the size of the training data.

In terms of computational cost, our approach is in between the “Cross-validation and testing” and “Nested cross-validation”. Firstly, each model with every combination of model parameters is trained *k* times in the *k*-fold cross-validation. And secondly, the model with the chosen combination of parameters is additionally trained *k*′ times in the *k*′-fold cross-testing.

We would also like to note that the data partitioning scheme proposed here is compatible with most machine learning models (including familiar classifiers and regressions). In the paper, we showed the method using SVM for illustration purposes.

It is our hope that the proposed approach can be a useful addition to the toolkit of machine learning approaches. We believe that it might be especially applicable when both data efficiency and parameter interpretations are desired.

## Supporting Information

S1 Public Repository We have included the full source code, graphs and data files for reproducibility. And also source files for the Figs 1, 2 and 3 which schematically explain different approaches. All the material can be accessed via Github repository: https://github.com/kristjankorjus/machine-learning-approaches.
(ZIP)
