# Supplementary figures and images for: An Efficient Data Partitioning to Improve Classification Performance While Keeping Parameters Interpretable
Source: PLoS One. 2016 Aug 26;11(8):e0161788. doi: 10.1371/journal.pone.0161788 (PMC5001642; doi:10.1371/journal.pone.0161788)

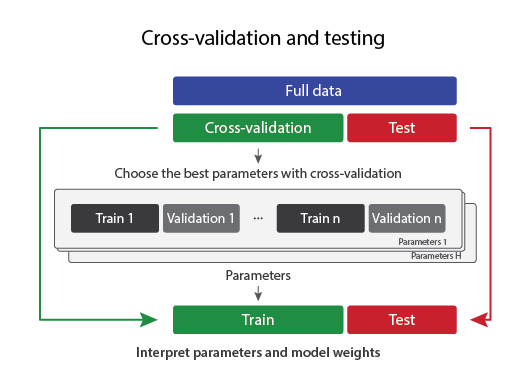

Supplement: S1 Public Repository — And also source files for the Figs 1, 2 and 3 which schematically explain different approaches. All the material can be accessed via Github repository: https://github.com/kristjankorjus/machine-learning-approaches. (ZIP) [file pone.0161788.s001.zip › machine-learning-approaches-master/final_figures/fig1.png]

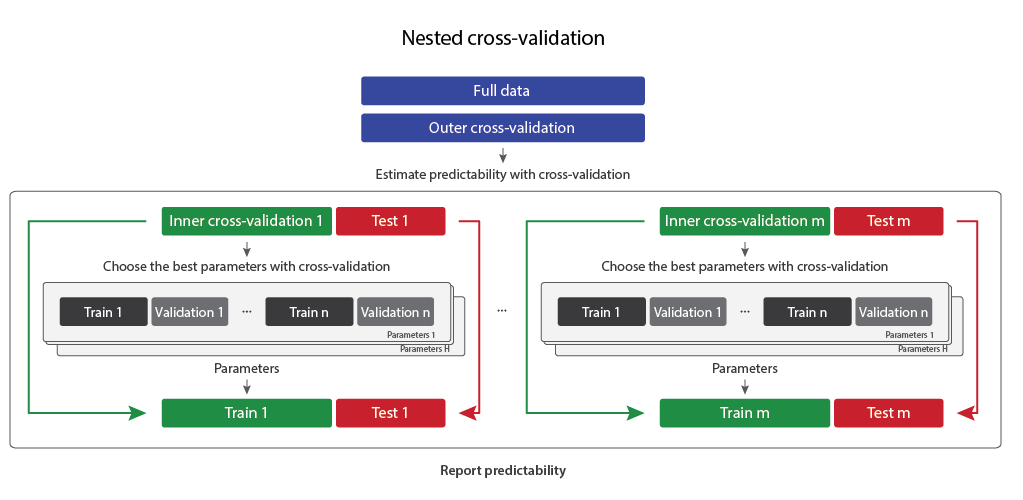

Supplement: S1 Public Repository — And also source files for the Figs 1, 2 and 3 which schematically explain different approaches. All the material can be accessed via Github repository: https://github.com/kristjankorjus/machine-learning-approaches. (ZIP) [file pone.0161788.s001.zip › machine-learning-approaches-master/final_figures/fig2.png]

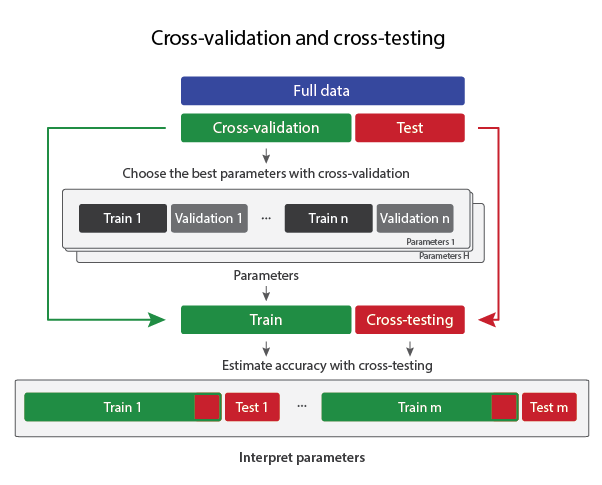

Supplement: S1 Public Repository — And also source files for the Figs 1, 2 and 3 which schematically explain different approaches. All the material can be accessed via Github repository: https://github.com/kristjankorjus/machine-learning-approaches. (ZIP) [file pone.0161788.s001.zip › machine-learning-approaches-master/final_figures/fig3.png]

# Simulated data

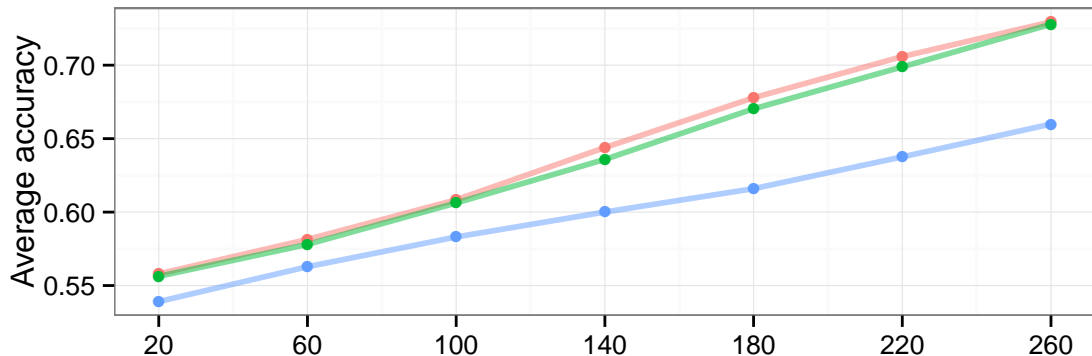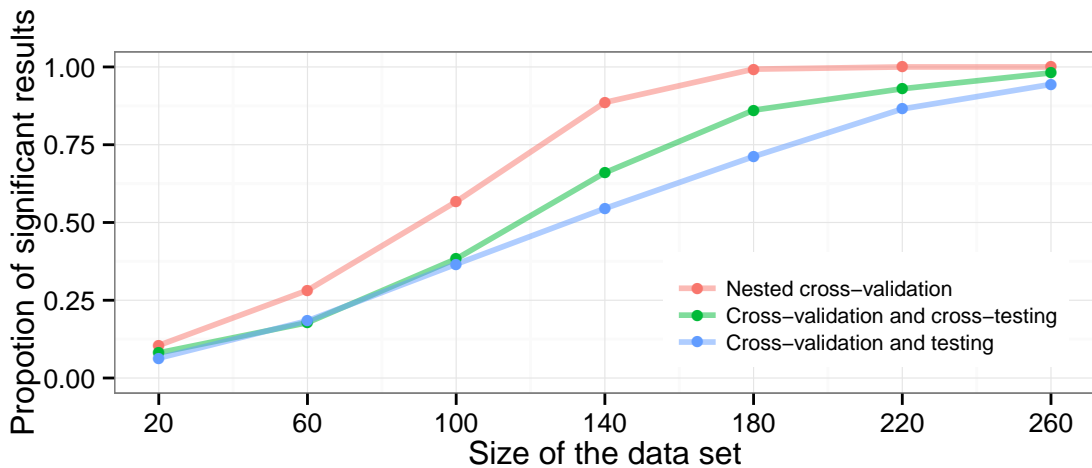

Supplement: S1 Public Repository — And also source files for the Figs 1, 2 and 3 which schematically explain different approaches. All the material can be accessed via Github repository: https://github.com/kristjankorjus/machine-learning-approaches. (ZIP) [file pone.0161788.s001.zip › machine-learning-approaches-master/final_figures/fig4.pdf]

# Simulated data

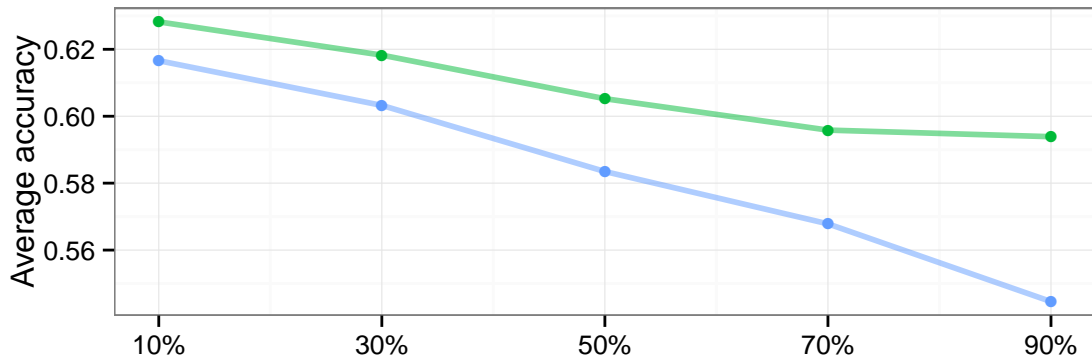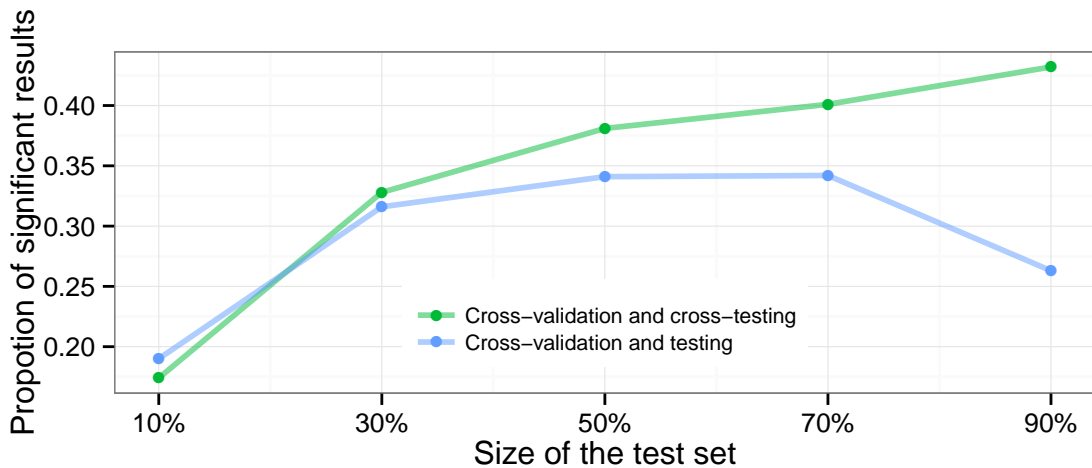

Supplement: S1 Public Repository — And also source files for the Figs 1, 2 and 3 which schematically explain different approaches. All the material can be accessed via Github repository: https://github.com/kristjankorjus/machine-learning-approaches. (ZIP) [file pone.0161788.s001.zip › machine-learning-approaches-master/final_figures/fig5.pdf]

# Electroencephalogram data

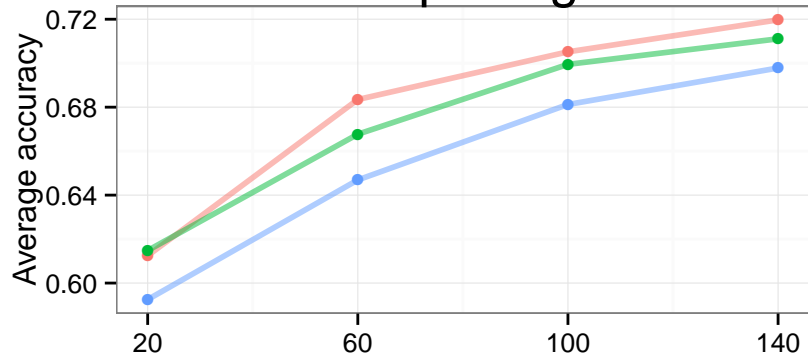

# Spikes data

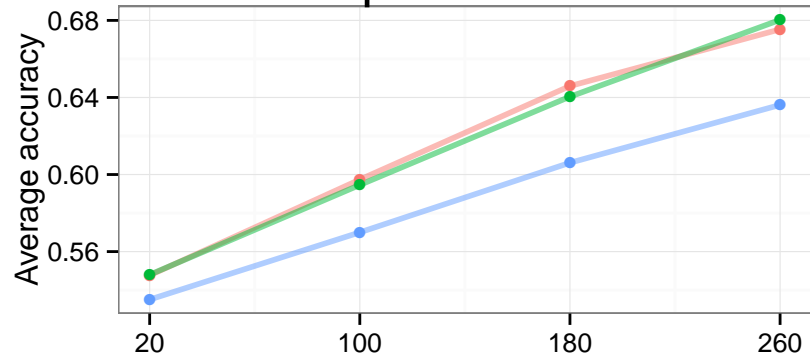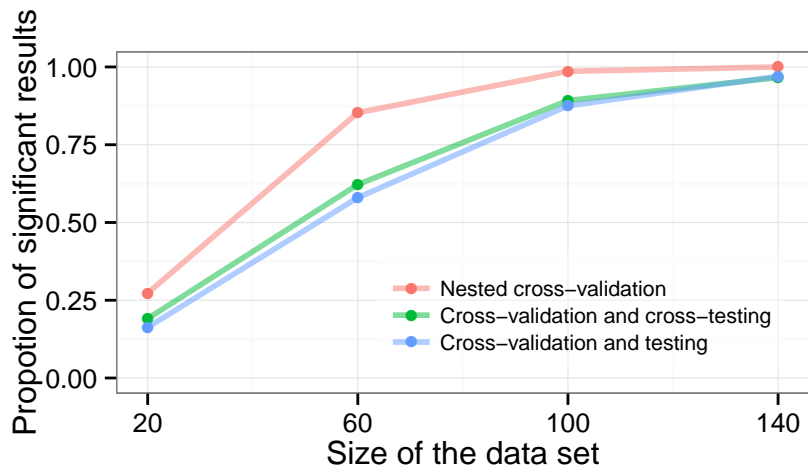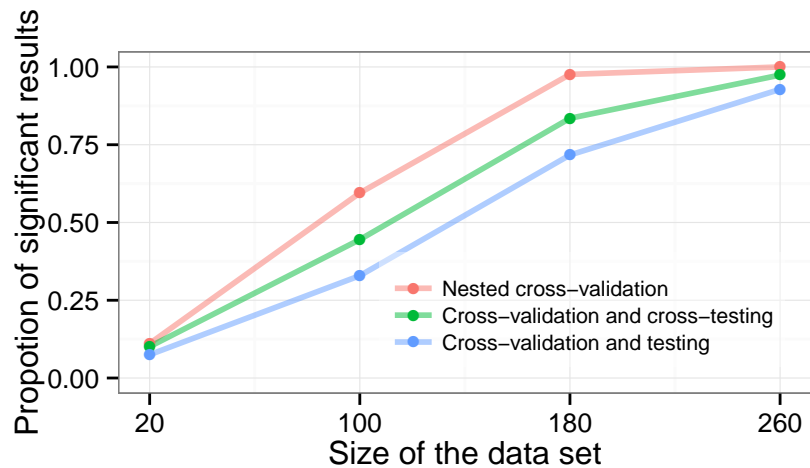

Supplement: S1 Public Repository — And also source files for the Figs 1, 2 and 3 which schematically explain different approaches. All the material can be accessed via Github repository: https://github.com/kristjankorjus/machine-learning-approaches. (ZIP) [file pone.0161788.s001.zip › machine-learning-approaches-master/final_figures/fig6.pdf]

# Electroencephalogram data

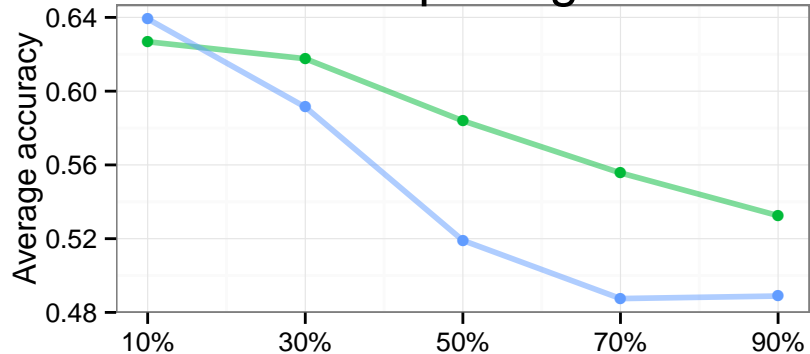

# Spikes data

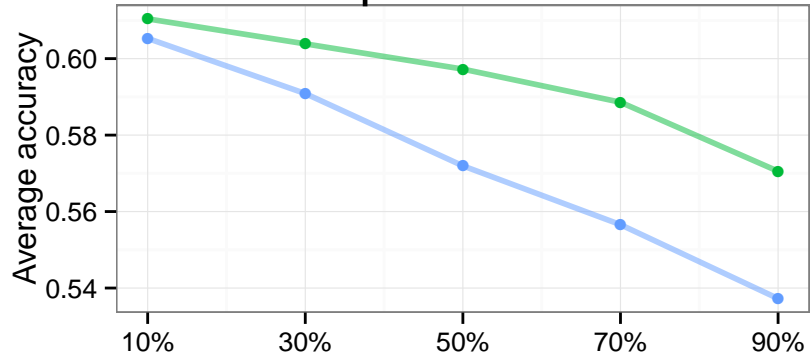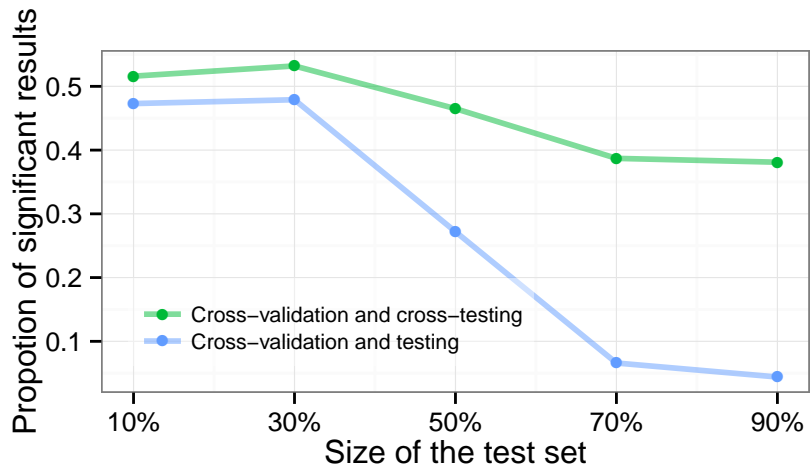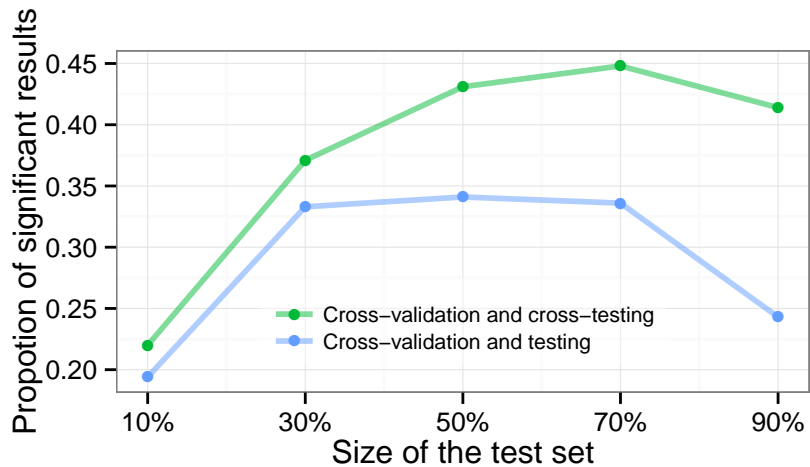

Supplement: S1 Public Repository — And also source files for the Figs 1, 2 and 3 which schematically explain different approaches. All the material can be accessed via Github repository: https://github.com/kristjankorjus/machine-learning-approaches. (ZIP) [file pone.0161788.s001.zip › machine-learning-approaches-master/final_figures/fig7.pdf]

# Random data

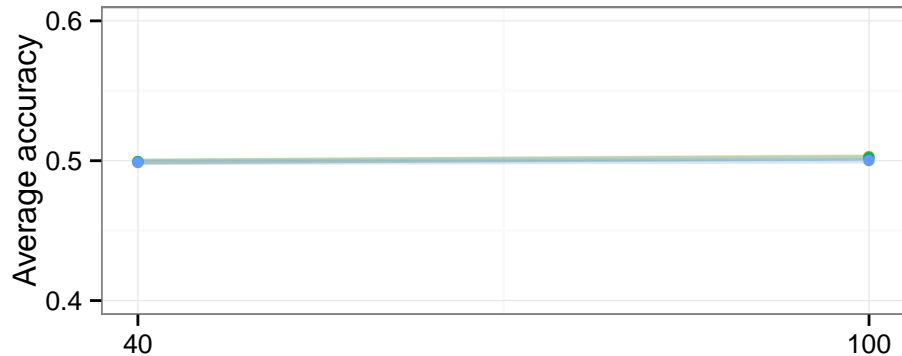

# Random data

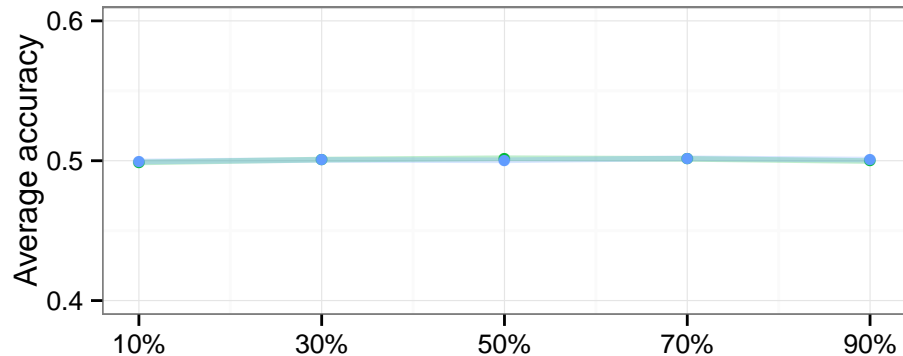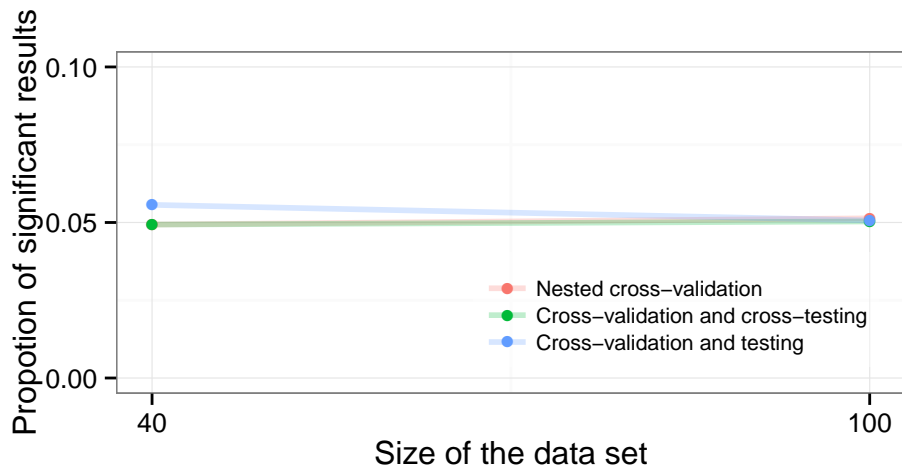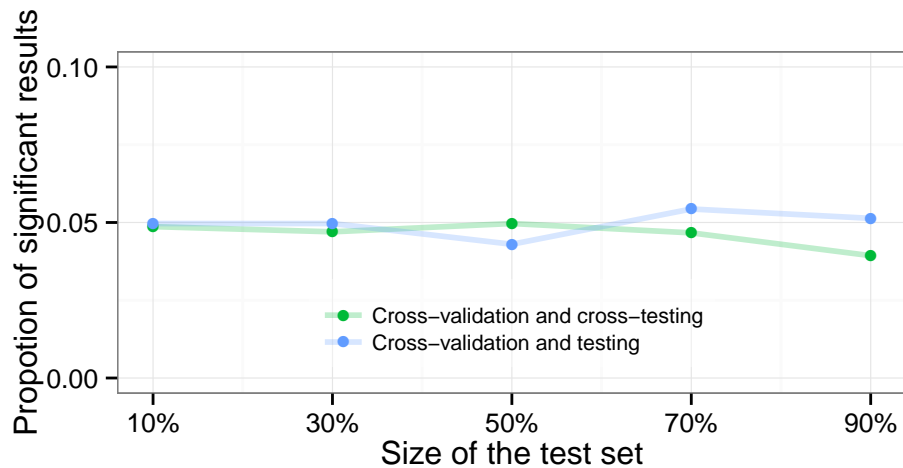

Supplement: S1 Public Repository — And also source files for the Figs 1, 2 and 3 which schematically explain different approaches. All the material can be accessed via Github repository: https://github.com/kristjankorjus/machine-learning-approaches. (ZIP) [file pone.0161788.s001.zip › machine-learning-approaches-master/final_figures/fig8.pdf]
